# Bmi-1 alleviates adventitial fibroblast senescence by eliminating ROS in pulmonary hypertension

**DOI:** 10.1186/s12890-021-01439-0

**Published:** 2021-03-05

**Authors:** Kai Li, Yan Li, Youjia Yu, Jingjing Ding, Huijie Huang, Chunyan Chu, Li Hu, Yanfang Yu, Yue Cao, Peng Xu, David Fulton, Feng Chen

**Affiliations:** 1 Department of Forensic Medicine, Nanjing Medical University, 101 Longmian Avenue, Nanjing, Jiangsu 211166 People’s Republic of China; 2 Vascular Biology Center, Medical College of Georgia at Augusta University, Augusta, Georgia; 3 Key Laboratory of Targeted Intervention of Cardiovascular Disease, Collaborative Innovation Center for Cardiovascular Disease Translational Medicine, Nanjing Medical University, Nanjing, Jiangsu 211166 People’s Republic of China

**Keywords:** Pulmonary hypertension, Senescence, Bmi-1, Lung fibroblast, Oxidative stress

## Abstract

**Objectives:**

Pulmonary hypertension (PH) is a life-threatening progressive disease with high mortality in the elderly. However, the pathogenesis of PH has not been fully understood and there is no effective therapy to reverse the disease process. This study aims to determine whether cellular senescence is involved in the development of PH.

**Methods:**

The rat PH model was established by intraperitoneal injection of monocrotaline and evaluated by pulmonary arteriole wall thickness and right ventricular hypertrophy index. Human lung fibroblasts (HLFs) were treated with CoCl_2_ or hypoxia to induce cellular senescence in vitro. SA-β-gal staining and the changes of senescent markers were used to examine cellular senescence. The molecular mechanism of cellular senescence was further explored by detecting reactive oxygen species (ROS) levels and culturing cells with a conditioned medium.

**Results:**

We revealed the cellular senescence of pulmonary adventitial fibroblasts in vivo in the rat PH model. The expression of Bmi-1, an important regulator of senescence, was decreased in the lungs of PH rats and localized in adventitial fibroblasts. The in vitro experiments showed that p16 expression was increased while Bmi-1 expression was decreased after CoCl_2_ treatment in HLFs. Mechanistically, Bmi-1 could alleviate CoCl_2_-induced HLFs senescence by eliminating ROS which further promoted the proliferation of pulmonary artery smooth muscle cells by paracrine mode of action of HLFs.

**Conclusion:**

Bmi-1 alleviates the cellular senescence of pulmonary fibroblasts in PH, which expands the pathogenesis of PH and provides a theoretical basis for targeting senescent cells in the treatment of PH.

**Supplementary Information:**

The online version contains supplementary material available at 10.1186/s12890-021-01439-0.

## Introduction

Pulmonary hypertension (PH) is a life-threatening progressive disease with elevated pulmonary artery pressures caused by various conditions. Hemodynamic diagnosis of PH is based on the mean pulmonary artery pressure ≥ 20 mmHg on resting right heart catheterization [1–3]. Pathology of PH is characterized by activated vasoconstriction and severe vascular remodeling. PH prevalence accounts for about 1% of the global population, but rises to 10% among people over the age of 65 with a marked increase in mortality, suggesting that PH is more common in the elderly who are prone to age-related pulmonary vascular disease [1, 4]. Moreover, age has been identified as an independent risk factor for death in patients with PH [1, 3, 4]. However, the pathogenesis of PH has not been fully elucidated. Recent researches suggested that pulmonary vascular remodeling results from multiple factors including genetic mutation (e.g. BMPR2), epigenetic modification (DNA methylation, histone acetylation, microRNAs, etc.) and pathogenic factors (hypoxia, infections, drugs or poisons, etc.) [5]. Whether age-related physiological and pathological senescence is one of the contributors of PH deserves an in-depth study.

Cellular senescence is a cell state triggered by a variety of stressors, such as genotoxic agents, nutritional deficiencies, hypoxia, mitochondrial dysfunction, and oncogene activation [6, 7]. It is characterized by a prolonged and generally irreversible cell-cycle arrest, apoptosis resistance, and abnormal expression of senescence-associated secretory phenotype (SASP) factors (IL-6, IL-8, IFN-γ, etc.) under various physiological processes and a wide spectrum of age-related disease conditions [8]. The increased activity of SA-β-gal and elevated expression of p16, p19, p21, and p53, as well as the SASP factors, are considered as the main markers of cellular senescence [7, 9–11]. Studies have revealed that cellular senescence can prevent or inhibit the proliferation of damaged or dysfunctional cells, acting as a protective role in disease development [9, 12]. However, the aberrantly accumulated senescent cells in tissues may lead to negative effects, including excessive secretion of SASP factors that affect the proliferation and migration of neighboring cells through the paracrine mode of action [10]. A lot of diseases, such as pulmonary fibrosis, atherosclerosis, and chronic obstructive pulmonary disease, have been linked to cellular senescence [7, 13–16]. Currently, researches have also shown that PH and cellular senescence share common triggers and pathogenic pathways, similar markers, and consistent intervention targets in vivo, suggesting the potential link between PH and cellular senescence [17]. However, so far, few studies have explored the effect of cellular senescence on PH, and the molecular mechanism underlying which is still unknown.

Previous literature suggests that PH has tumor-like characteristics, and many proto-oncogenes are involved in the phenotypic changes [18]. B-lymphoma MO-MLV insertion region 1 (Bmi-1), a member of the multi-comb family of transcription repressors, is a proto-oncogene and regulates cell cycle and cellular senescence via inhibition of p16^INK4a^/Rb and p19^ARF^/p53 pathways [19–23]. Since its discovery, Bmi-1 is reported to be involved in many biological pathways, including organ development, cell cycle, DNA damage response (DDR), senescence, stem cells, and self-renewal [23–28]. Some of them are the known causes of PH. Thus, we wondered whether cellular senescence is involved in the development of PH.

The purpose of this study was to determine the role of cellular senescence in the development of PH and figure out whether Bmi-1 was involved in the process. Via monocrotaline (MCT) induced PH model in vivo and hypoxia/CoCl_2_ induced cellular senescence models in vitro, we observed the enhanced lung adventitial fibroblasts senescence in PH and further explored the role of Bmi-1 in the pathogenesis of PH.

## Methods

### Animal models

PH was induced in rats by MCT (Sigma-Aldrich, St. Louis, MO, USA) [29, 30]. Adult male Sprague–Dawley rats (250–300 g) were randomly divided into the MCT group and control group (n = 8 per group). Rats in the MCT group were injected with a single dose of MCT (60 mg/kg, i.p.) which elicited a progressive, severe and irreversible form of PH after 2–4 weeks. Rats in the control group were injected with the same volume of vehicle. Rats were housed at constant temperature (21–23 °C) with free access to food and water and 12 h light–dark cycles. After 4 weeks, rats were sacrificed and hearts and lungs were harvested for the subsequent experiments. Right ventricular hypertrophy was determined by normalizing the weight of the right ventricular to that of the left ventricular plus septal to calculate the RV/(LV+S), named Fulton index. All animal experiments were approved by the Institute for Laboratory Animal Research of Nanjing Medical University and the study was carried out in compliance with the ARRIVE guidelines.

### Histological, SA-β-gal, immunofluorescence and immunohistochemistry staining

Rat lung tissues were fixed in 4% paraformaldehyde, embedded in paraffin and then sliced into 5-μm-thick sections. Xylene was used for de-paraffinizing the paraffin-embedded sections, and a graded series of ethanol were used for processing the sections. Then, sections were stained with hematoxylin and eosin (H&E) sequentially. SA-β-gal staining was performed with Senescence β-Galactosidase Staining Kit (CST, Danvers, MA, USA) following the manufacturer’s instruction. The percentage of SA-β-gal positive cells was calculated and normalized to the control group. Immunofluorescence (IF) staining of rat lung tissue was performed as previously described [31]. The slides were stained with rabbit-anti-Bmi-1 (CST, 1:100 dilution), mouse-anti-α-SMA (CST, 1:100 dilution), mouse-anti-fibronectin (SANTA CRUZ, USA, 1:100 dilution), and DAPI (Sigma-Aldrich) and observed with fluorescence microscopy (OLYMPUS, Japan). Immunohistochemistry (IHC) staining of rat lung tissues was performed as previously described [32] and the slides were stained with fibronectin primary antibody (SANTA CRUZ, 1:50 dilution).

### Western blotting analysis

The RIPA (Sigma-Aldrich) buffer containing complete Protease Inhibitor Cocktail (Sigma-Aldrich) and PMSF (Beyotime, China) was used to extract proteins from lung tissues and cells. The extracts were centrifuged at 4℃ for 15 min at 12,000 rpm and the supernatants were quantified with the BCA protein assay kit (Beyotime). Equal amounts of total protein were separated by SDS-PAGE and transferred to PVDF membranes (Millipore, USA). Membranes were blocked with 5% skim milk at room temperature for 1 h and then incubated with the primary antibodies at 4℃ overnight. After washing with TBST (Tris-buffered saline, 0.1% Tween-20), the membranes were subsequently incubated with secondary antibodies for 1 h at room temperature. Protein bands were visualized with the Enhanced Chemiluminescence Detection Kit (Thermo Fisher) via the Tannon luminescent imaging system. Image J was used for the quantitative analysis of the protein bands. The antibodies used are listed below: rabbit-anti-Bmi-1(CST, 1:2000 dilution), rabbit-anti-p16 (Proteintech, USA, 1:1000), mouse-anti-β-actin (CST, 1:5000 dilution), rabbit-anti-GAPDH (CST, 1:5000 dilution), rabbit-anti-pH2A.X (Beyotime, 1:1000).[33].

### Quantitative real-time PCR analysis

Total RNA was isolated from lung tissues or cells using TRIzol (Invitrogen, USA) as described [34], and was reverse-transcribed into cDNA with 5 × All-In-One RT MasterMix with AccuRT Genomic DNA Removal Kit (abm, Canada) according to the manufacturer’s instructions. Relative gene expressions were determined using QSYBR Green qRT-PCR kit (Bio-Rad, USA) with the following primers: rat *Bmi-1*: CTGGATGCCAAGTGGTCTTT (forward), GCTGGTCTCCAAGTAACGCA (reverse); rat *β-actin*: CGCGAGTACAACCTTCTTGCAGGT (forward), CGTCATCCATGGCGAACTGG (reverse); rat *p16*: CGTACCCCGATACAGGTGATG (forward), ATACCGCAAATACCGCACGA (reverse); rat *p19*: GCCTTGCAGGTCATGATGTTT (forward), CCAGAGGCATCTTGGACGTT (reverse).

### Cell culture and treatment

The human pulmonary artery smooth muscle cell line (PASMC) and human lung fibroblast cell line (HLF) were purchased from Lonza (Swiss). PASMCs were cultured in SmGM-2 BulletKit media (Lonza) containing 5% fetal bovine serum (FBS), growth factors, and antibiotics. HLFs were maintained in Dulbecco’s modified Eagle’s medium (DMEM) with 10% FBS and antibiotics. Cells were incubated under 5% CO_2_ at 37℃. HLFs were transfected with Bmi-1 si-RNA (Ribobio, China) to silence its expression according to the manufacturer’s instructions. To build genetic overexpression of Bmi-1, Ad-CMV-human Bmi-1 (adv-Bmi-1) was purchased from TranSheep Bio (Shanghai, China). HLFs were infected with adv-Bmi-1 following the manufacturer’s instructions. Total cellular RNA was extracted to confirm the transfection efficiency by qRT-PCR.

For western blotting analysis, HLFs were cultured in the plate to confluence of 80%, and then washed with PBS and replaced with serum-free DMEM. CoCl_2_ (150 μmol/ml, Sigma-Aldrich) was added to the media for 0, 6, 12, 24 and 48 h and then cells were harvested for protein analysis. For reactive oxygen species (ROS) measurement, HLFs were infected with adv-Bmi-1 or transfected with si-Bmi-1, and then were treated with CoCl_2_ (150 μmol/ml) for 0, 6, 12, 24 and 48 h. For EDU assay, HLFs were exposed to hypoxia (1%O_2_, 5%CO_2_, and 1%FBS) for 96 h after infected with adv-Bmi-1 or transfected with si-Bmi-1. The supernatant of the cells following hypoxia exposure was filtered and added into the serum-free culture medium of PASMCs with equal volume as the conditioned medium (CM) for 48 h. The initial number of cells in each group was kept the same.

### EDU assays

PASMCs proliferation was detected by EDU kit (Beyotime). This assay was performed following the manufacturer’s instructions. Fluorescence was observed with fluorescence microscopy (OLYMPUS).

### ROS measurement

ROS was detected by Fluorometric Intracellular Ros Kit (Sigma-Aldrich) according to the manufacturer’s instructions and visualized with fluorescence microscopy (LIFE, USA). The red staining indicated the ROS-positive cells.

### Statistical analysis

Statistical analysis was performed using Graphpad Prism 7.00. An unpaired Student’s *t *test was used for single comparisons between two groups. Data are presented as the mean ± SEM. Significance was set as *P* < 0.05.

## Results

### Adventitial fibroblasts senescence is observed in MCT-induced PH rat model

**Fig. 1:**
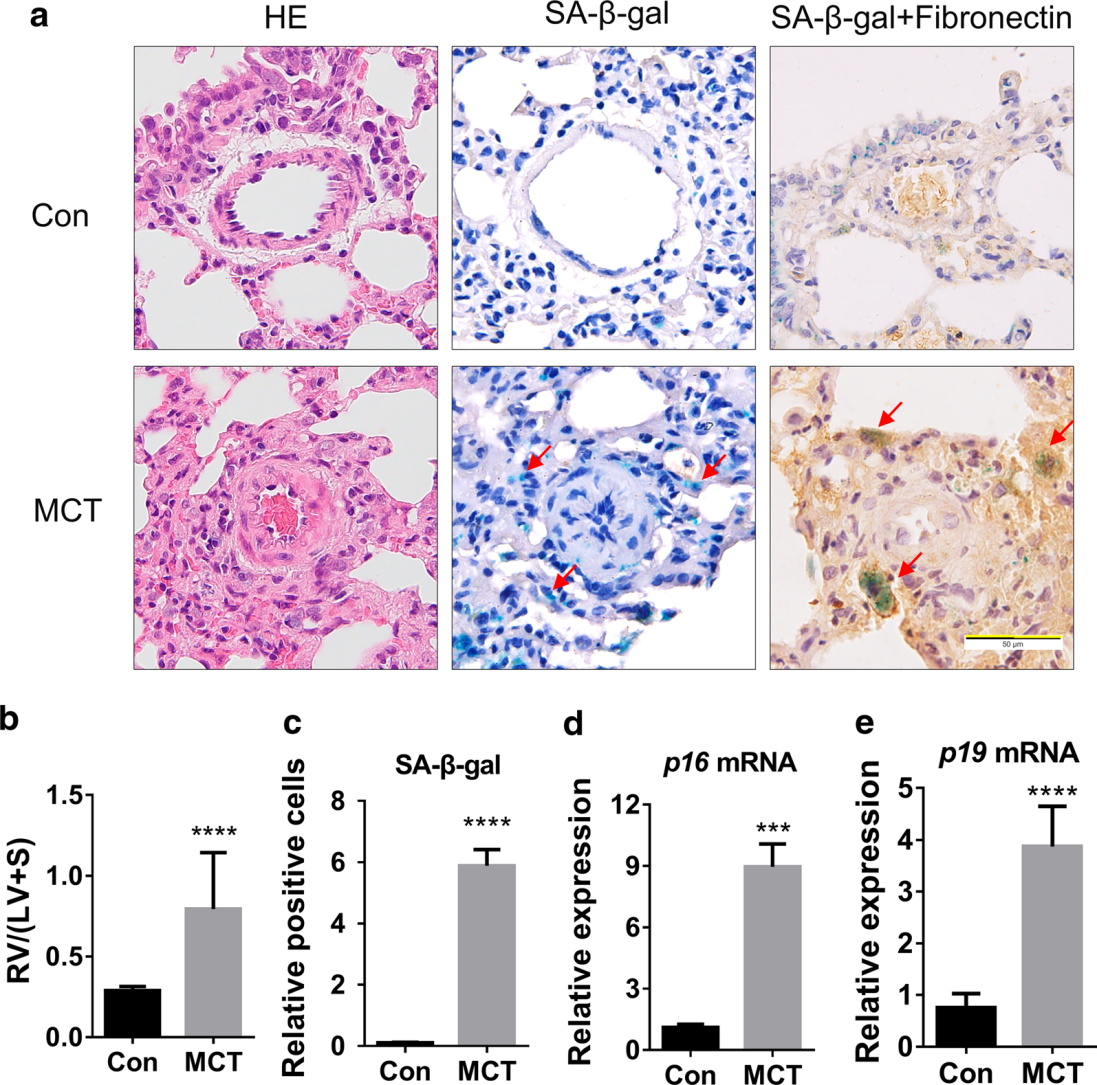
Adventitial fibroblasts senescence is observed in the MCT-induced PH rat model. **a**. Representative images of H&E staining, SA-β-gal staining and Fibronectin-IHC with SA-β-gal staining of rat lung tissues in MCT and control groups. **b** RV/(LV+S) was determined in each group (n = 8 rats per group). **c** The quantification of SA-β-gal positive cells normalized to the Con group in Fig. 1a (three pulmonary arteries per rat). **d**, **e**. Fold change by qRT-PCR: relative mRNA level of p16/β-actin and p19/β-actin in lung tissues of MCT rats to that in control rats (n = 3 rats per group). All data are shown as the mean ± SEM. Statistical significance compared to controls was assessed using the unpaired two-tailed Student’s *t* test: ***P < 0.001, ****P < 0.0001

To determine if cellular senescence was involved in PH lungs, we constructed an MCT-induced PH rat model. In this model, we observed thickened pulmonary arteriole walls and a significant elevated RV/(LV+S) ratio between the MCT group and the control group (Fig. 1a, b). SA-β-gal staining showed that the number of SA-β-gal positive cells in the MCT group was significantly greater than that in the control group (Fig. 1a, c). Interestingly, the SA-β-gal positive cells were mainly located in the adventitia matrix. To examine the type of senescent cells, we stained fibronectin by IHC after SA-β-gal staining. Results showed the co-localization of SA-β-gal and fibronectin in the adventitia matrix of the pulmonary artery (Fig. 1a), Which indicated that the fibroblasts around pulmonary arterioles were the prominent senescent cell type in PH. Meanwhile, compared with the control group, SA-β-gal and fibronectin co-stained cells in the pulmonary arteriole matrix of the MCT group were larger in volume, more irregular in morphology, and stacked together. These changes coincided with the characteristics of senescent cells.

Next, we tested the expression of the markers of cellular senescence, including p16 and p19, to verify tissue senescence. As expected, the expression levels of *p16* and *p19* mRNA in lung tissues of MCT group were significantly higher compared with those of the control group (Fig. 1d, e). Since DNA damage existed in both PH progression and cell senescence processes, we detected the level of γ-H2A.X protein (a protein that represents DDR). We found that the levels of γ-H2A.X protein in lung tissues of MCT group were significantly higher than that in the control group (Additional file 1: Fig. S1). These results confirmed lung tissue senescence in the MCT-induced PH rat model and demonstrated that pulmonary adventitial fibroblasts are the main cell type involved in cellular senescence.

### Bmi-1 is mainly expressed in pulmonary adventitial fibroblasts

**Fig. 2:**
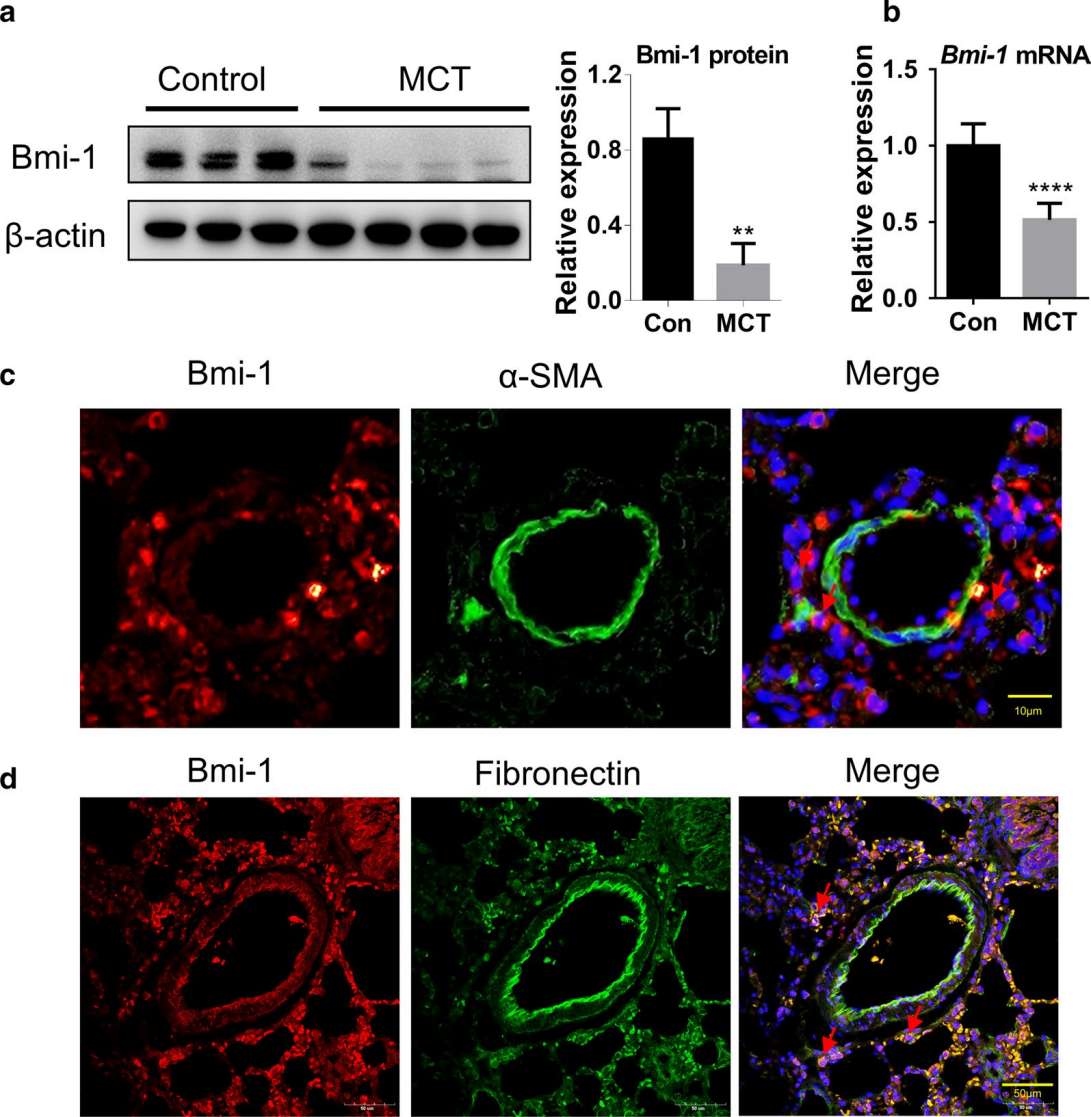
Bmi-1 is mainly expressed in pulmonary adventitial fibroblasts and its expression is reduced in PH rat model. **a** Western blotting of Bmi-1 from rat lung tissues of control (n = 3) and MCT group (n = 4). **b** qRT-PCR results of relative mRNA level of *Bmi-1/*/*β-actin* in lung tissues of each group (n = 3 rats per group). **c** IF staining for α-SMA and Bmi-1 in the rat lung tissue. Red represents Bmi-1 staining, green represents α-SMA staining, blue represents nuclear DNA staining by DAPI. **d** IF staining for fibronectin and Bmi-1 in the rat lung tissue. Red represents Bmi-1 staining, green represents fibronectin staining, blue represents nuclear DNA staining by DAPI. All data are shown as the mean ± SEM. Statistical significance was assessed using the unpaired two-tailed Student’s *t* test: **P < 0.01, ****P < 0.0001

Bmi-1 regulates cell cycle and senescence via inhibition of p16^INK4a^/Rb and p19^ARF^/p53 pathways, and it also mediates cellular redox balance [19, 21]. Thus, we detected the expression of Bmi-1 in the lung tissues of the MCT-induced PH rat model. The protein expression and mRNA levels of Bmi-1 in lung tissues of the MCT group were significantly lower than those of the control group (Fig. 2a, b), which suggested that Bmi-1 might participate in the development of PH.

To determine the cellular localization of Bmi-1, we co-stained α-smooth muscle actin (α-SMA) and Bmi-1 in the rat lung tissues by IF staining. We found that the localization of Bmi-1 was inconsistent with the expression pattern of α-SMA, indicating that Bmi-1 was not expressed in the media smooth muscle cells (Fig. 2c). Then, we co-stained Bmi-1 and fibroblast marker fibronectin and observed that Bmi-1 and fibronectin were co-localized in the extravascular matrix of PH lungs (Fig. 2d). These results suggest that Bmi-1 is mainly expressed in pulmonary adventitial fibroblasts.

### Bmi-1 directly regulates HLFs senescence by eliminating ROS

**Fig. 3:**
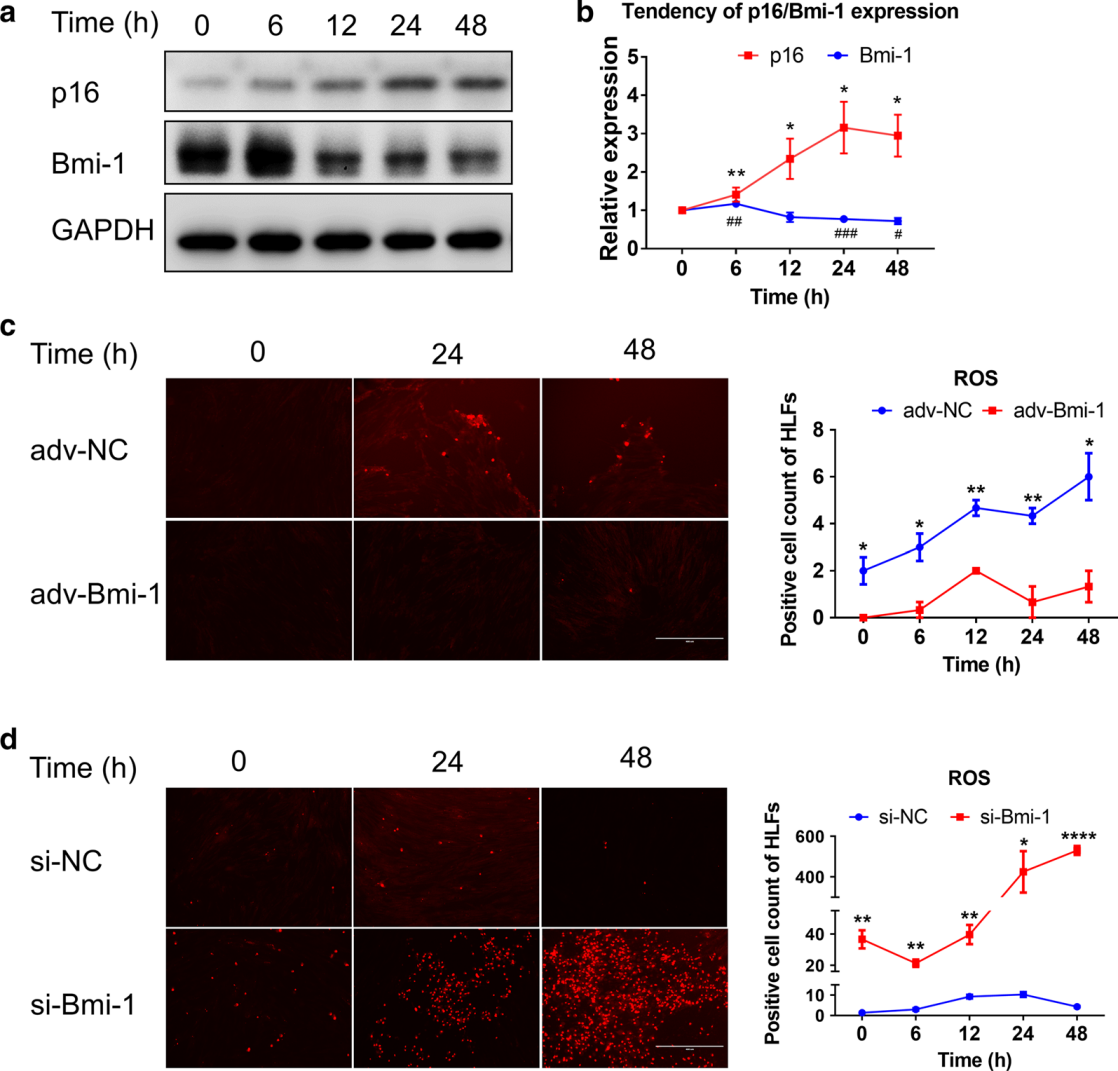
Bmi-1 directly regulates HLFs senescence by eliminating ROS. HLFs were given CoCl_2_ (150 μmol/ml) for 0, 6, 12, 24, and 48 h. **a** Western blotting detected the expression of Bmi-1 and p16 in each group. **b** Expression trend of Bmi-1 and p16 protein at 0, 6, 12, 24, and 48 h. *P < 0.05 and **P < 0.01 versus 0 h of p16 group. ^#^*P* < 0.05, ^##^*P* < 0.01 and ^###^*P* <0.001 vs. 0 h of Bmi-1 group. **c**, **d** Detection of the number of ROS-positive HLFs in adv-Bmi-1 or si-Bmi-1 group after each group was treated with CoCl_2_ for 0, 6, 12, 24, and 48 h. *P < 0.05, **P < 0.01, and ****P < 0.0001 vs. adv-NC (**c**) or si-NC (**d**) group in indicated time respectively. All data are shown as the mean ± SEM of at least three independent experiments. Statistical significance compared to controls was assessed using the unpaired two-tailed Student’s *t* test

We then explored if fibroblasts senescence was regulated by the *Bmi-1* gene. HLFs were given CoCl_2_ (150 μmol/ml) for 0, 6, 12, 24, and 48 h. Compared with the 0 h control group, the expression of Bmi-1 protein was slightly increased in the initial 6 h group, and decreased in the rest groups (Fig. 3a). However, the expression of p16 protein was increased in all treatment groups (Fig. 3a). On the trend chart, after 6 h, opposing expression trends of Bmi-1 and p16 were observed (Fig. 3b). This result indicated that Bmi-1 expression in HLFs was increased early when stimulated with pathogenic factors, but decreased gradually with enhanced cellular senescence indicated by increased p16 expression.

ROS is considered to be one of the causes of PH and one of the factors leading to senescence [17, 34–36]. Bmi-1 can mediate the ROS levels and maintain redox balance in cells [25–27]. To explore whether Bmi-1 could also mediate cellular senescence by modulating the ROS levels of HLFs in PH, we overexpressed or silenced Bmi-1 in HLFs by the Bmi-1 adenovirus (adv-Bmi-1) or siRNA, respectively. The transfection efficiencies of si-Bmi-1 and adv-Bmi-1 were confirmed by qRT-PCR (Additional file 1: Fig. S2). HLFs were treated with CoCl_2_ for 0, 6, 12, 24, and 48 h after infection by adenovirus or transfection with siRNA, respectively. The number of ROS-positive HLFs in each group was determined. The results showed that ROS positive cells in the adv-Bmi-1 group were more in number than that in the control group (Fig. 3c), but which in the si-Bmi-1 group was less than that in the control group (Fig. 3d). Moreover, after infected with adv-Bmi-1 or transfected with si-Bmi-1, HLFs were also cultured under hypoxic conditions (1%O_2_, 5%CO_2_, and 1%FBS) for 96 h. Cellular senescence was further detected by SA-β-gal staining in each group. It showed that the number of SA-β-gal positive cells were increased significantly in the si-Bmi-1 group. However, when the Bmi-1 expression was increased, the number of SA-β-gal positive cells was not changed significantly, and the cell morphology was normal (Additional file 1: Fig. S3). These results suggested that Bmi-1 regulates the senescence of HLFs partially by eliminating ROS.

### Fibroblasts Bmi-1 alters PASMC proliferation by paracrine mode of action

**Fig. 4:**
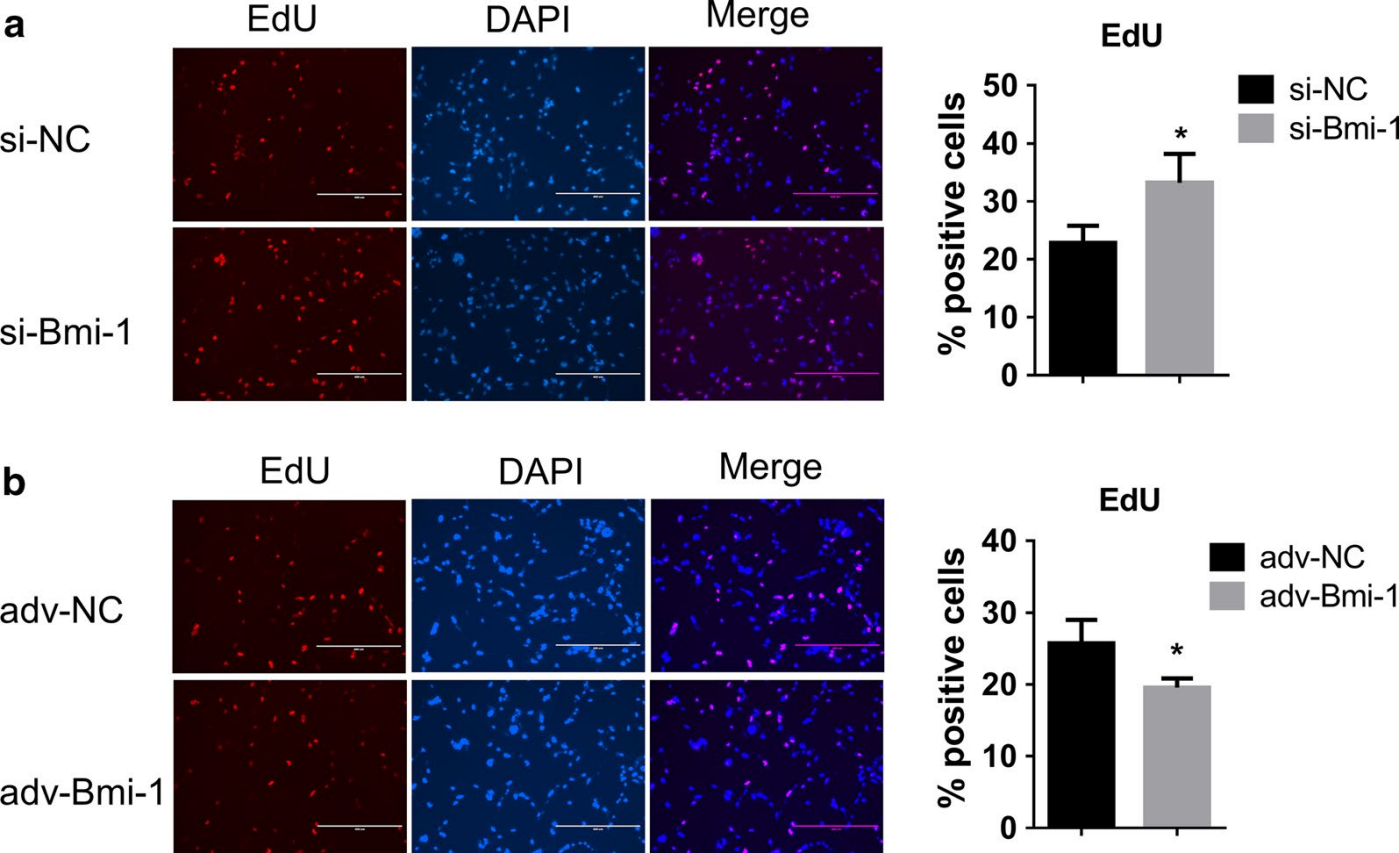
Fibroblasts Bmi-1 alters PASMC proliferation by paracrine mode of action. **a**–**b**. EDU staining for PASMCs treated with the CM of HLFs. HLFs were infected with adv-Bmi-1 or transfected with si-Bmi-1 following by hypoxia exposure for 96 h, and then the supernatant was collected and added the equal volume of serum-free culture medium to culture PASMCs for 48 h. All data are shown as the mean ± SEM of at least three independent experiments. Statistical significance was assessed using the unpaired two-tailed Student’s t test: *P < 0.05

It has been reported that senescent fibroblasts secreted SASP factors into the extracellular matrix (ECM), which could be able to regulate the function of adjacent cells [15, 37, 38]. To explore the effect of senescent fibroblasts on the adjacent vascular smooth cells, we collected the supernatant of fibroblasts with adv-Bmi-1 infection or si-Bmi-1 transfection following by hypoxia treatment for 96 h. Then, the supernatant of each group was added into the serum-free culture medium of PASMCs with equal volume as the CM for 48 h. The EDU staining showed more positive cells in the group with the CM of HLFs treated with si-Bmi-1 (Fig. 4a), while less EDU positive PASMCs cultured with the CM of HLFs treated with adv-Bmi-1 (Fig. 4b), indicating that the SASP factors of lung fibroblasts induced by the silenced Bmi-1 promoted the PASMCs proliferation. These results suggested that lung fibroblasts Bmi-1 alters PASMC proliferation by paracrine mode of action.

## Discussion

As a severe degenerative cardiovascular disease in the elderly, PH is typically characterized by significant pulmonary arteriolar remodeling and right heart failure. Previous studies have found that the remodeling of the vascular wall is mainly caused by the proliferation and migration of the PASMCs, and these changes occur in the early process of PH [18, 39, 40]. However, when the inflammatory cells are continuously recruited, the inflammation gradually aggravates. Perivascular inflammation is one of the main phenotypes of end-stage pulmonary vascular, contributing to the irreversible PH [5, 17, 41]. Mechanistically, this characteristic of PH accords with the function of senescent cells, that is, the senescent cells that accumulate in the tissue gap cannot be removed in time and will continuously secrete SASP factors, resulting in the recruitment of immune cells and the persistence of tissue inflammation [12, 42]. The latest study reveals that cellular senescence impairs the reversibility of PH by MCT+ shunt-induced PH rat model and confirms that pulmonary endothelial cells of patients with PAH are more vulnerable to senescence than controls in response to shear stress [43]. Here, we also verify the existence of cellular senescence in the PH rat model and further indicate that the pulmonary adventitial fibroblast is the main senescent cell type.

To explore the mechanism of fibroblast senescence, we focused on Bmi-1, which is a proto-oncogene that mainly mediates oxidative stress and DDR, cell self-renewal, and senescence [20, 25, 26]. We found that the expression of Bmi-1 is reduced in PH and localized in adventitial lung fibroblasts. In vitro experiments showed that the protein levels of Bmi-1 increased in the early stage of hypoxia treatment, but gradually decreased with the time prolonged. Moreover, Bmi-1 was confirmed to alleviate fibroblasts' senescence by eliminating ROS. These results prompted us to hypothesize that in the early stages of PH formation, Bmi-1 would be activated as the stimuli causing DNA damage, increased ROS concentration, and abnormal mitochondrial function. The activated Bmi-1 would be beneficial in vascular wall homeostasis and tissue repair. However, as the disease progressing, the expression of Bmi-1 might be decreased and the number of senescent fibroblasts would increase, forcing PH to another direction with persistent chronic inflammation. Unlike previous reports, this might be a new mechanism that the senescence of adventitial fibroblasts could be involved in the pathophysiology of PH.

Studies have demonstrated that the fibroblasts in the pulmonary vascular adventitia are one of the contributing cell types of PH, which act by directly or indirectly differentiating into myofibroblasts or through the recruitment of circulating inflammatory cells [38, 44–47]. Fibroblasts can regulate the elements of ECM, and can also secrete growth factors, cytokines, and chemokines [44, 48]. It not only senses and guides the response to a variety of stimuli, but also communicates with smooth muscle cells and endothelial cells by paracrine mode of action [44, 45, 49]. Moreover, under the action of external stimulation during PH progression, senescent fibroblasts can secrete SASP factors into the ECM, which involves a variety of inflammatory factors that induce an inflammatory response in adjacent tissues [7, 9, 11, 50, 51]. In our research, we revealed that overexpression of Bmi-1 in HLFs inhibited cellular senescence and reduced the proliferation of PASMCs, suggesting that Bmi-1 altered PASMC proliferation indirectly by mediating HLF secretions.

Based on all of the above results, we have made a hypothesis on the pathological mechanism of PH from the perspective of cellular senescence, which enriches the understanding of PH from a different angle. This hypothesis is: (1) The pathological stimuli in PH causes stress injury and dysfunction of pulmonary arterial cells; (2) DNA damage repair is subsequently activated, which stimulates proto-oncogene Bmi-1 to protect vascular remodeling by eliminating ROS in the early stage; (3) As the existence of persistent stimuli, the expression of Bmi-1 is gradually reduced and the senescence of adventitial fibroblasts are induced; (4) Senescent adventitial fibroblasts release SASP factors to aggravate the inflammatory responses, which promote the proliferation of smooth muscle cells, and finally lead to the vascular muscularization. Based on the higher degree of physiological cellular senescence in the elderly population, the enhanced SASP factors further exacerbate PH progression, which may be the reason why there are higher incidence and mortality of PH in the elderly [1, 4, 52].

## Conclusions

Here, using an MCT-induced PH rat model, we demonstrate the existence of cellular senescence of pulmonary adventitial fibroblasts in PH. We reveal that the fibroblast senescence is alleviated by Bmi-1 through eliminating ROS and the senescent fibroblasts can promote PASMC proliferation by paracrine mode of action. Our research provides novel insight into the course of PH from the perspective of senescence, lays a foundation for further study of the role of senescence in PH, and serves as proof of the concept that the prevention and elimination of cellular senescence can be a novel strategy in the treatment of PH.

## Supplementary Information

**Additional file 1.** **Figure S1.** Expression of γ-H2A.X in MCT-induced PH rat model. A. Western blotting of γ-H2A.X from rat lung tissues of the contrl (n=4) and MCT group (n=4). B. Quantification of the protein expression of γ-H2A.X in the two groups. All data are shown as the mean ± SEM. Statistical significance compared to controls was assessed using the unpaired two-tailed Student’s *t*-test: *** *P* < 0.001. **Figure S2.** Transfection efficiency detection. The transfection efficiency of adv-Bmi-1 (A) and si-Bmi-1 (B) in HLFs. All data are shown as the mean ± SEM. Statistical significance compared to controls was assessed using the unpaired two-tailed Student’s *t*-test: **** *P* < 0.0001. **Figure S3.** SA-β-gal staining of HLFs treated with adv-Bmi-1 and si-Bmi-1. A. Respresentitive images. Red arrows indicate the positively stained cells. B. The quantification of SA-β-gal positive cells are on the right. Three visual fields were randomly selected and the number of positive cells versus the total number of cells in each field was counted. All data are shown as the mean ± SEM. Statistical significance compared to controls was assessed using the unpaired two-tailed Student’s t-test: ** *P* < 0.01.

## Data Availability

The datasets used and/or analyzed during the current study are available from the corresponding author on reasonable request.

## Abbreviations

Bmi-1: B-lymphoma MO-MLV insertion region 1
CM: Conditioned medium
DDR: DNA damage response
ECM: Extracellular matrix
HLFs: Human lung fibroblasts
IF: Immunofluorescence
MCT: Monocrotaline
PASMCs: Pulmonary artery smooth muscle cells
PH: Pulmonary hypertension
ROS: Reactive oxygen species
SASP: Senescence-associated secretory phenotype
α-SMA: α-Smooth muscle actin
