# Supplementary material for: Bmi-1 alleviates adventitial fibroblast senescence by eliminating ROS in pulmonary hypertension
Source: BMC Pulm Med. 2021 Mar 5;21:80. doi: 10.1186/s12890-021-01439-0 (PMC7934412; doi:10.1186/s12890-021-01439-0)
Supplement: Supplementary file 1 — Additional file 1. Figure S1. Expression of γ-H2A.X in MCT-induced PH rat model. A. Western blotting of γ-H2A.X from rat lung tissues of the contrl (n=4) and MCT group (n=4). B. Quantification of the protein expression of γ-H2A.X in the two groups. All data are shown as the mean ± SEM. Statistical significance compared to controls was assessed using the unpaired two-tailed Student’s t-test: *** P < 0.001. Figure S2. Transfection efficiency detection. The transfection efficiency of adv-Bmi-1 (A) and si-Bmi-1 (B) in HLFs. All data are shown as the mean ± SEM. Statistical significance compared to controls was assessed using the unpaired two-tailed Student’s t-test: **** P < 0.0001. Figure S3. SA-β-gal staining of HLFs treated with adv-Bmi-1 and si-Bmi-1. A. Respresentitive images. Red arrows indicate the positively stained cells. B. The quantification of SA-β-gal positive cells are on the right. Three visual fields were randomly selected and the number of positive cells versus the total number of cells in each field was counted. All data are shown as the mean ± SEM. Statistical significance compared to controls was assessed using the unpaired two-tailed Student’s t-test: ** P < 0.01. [file 12890_2021_1439_MOESM1_ESM.docx]

**Bmi-1 alleviates adventitial fibroblast senescence by eliminating ROS in pulmonary hypertension**

Kai Li^a,1^, Yan Li ^a,1*^, Youjia Yu^a,1^, Jingjing Ding^a^, Huijie Huang^a^, Chunyan Chu^a^, Li Hu^a^, Yanfang Yu^a^, Yue Cao^a^, Peng Xu ^a^, David Fulton ^b^, Feng Chen^a,c*^

^a^ Department of Forensic Medicine, Nanjing Medical University, Nanjing, Jiangsu, 211166, P.R. China.

^b^ Vascular Biology Center, Medical College of Georgia at Augusta University, Augusta, Georgia.

^c^ Key Laboratory of Targeted Intervention of Cardiovascular Disease, Collaborative Innovation Center for Cardiovascular Disease Translational Medicine, Nanjing Medical University, Nanjing, Jiangsu, 211166, People's Republic of China.

^1^ These authors contributed equally to this work.

^*^Correspond to:

Yan Li, Ph.D.

Department of Forensic Medicine, Nanjing Medical University,

101 Longmian Avenue, Nanjing, Jiangsu 211166, China.

E-mail: liyan_njmu@163.com

Feng Chen, M.D. & Ph.D.

Department of Forensic Medicine, Nanjing Medical University,

101 Longmian Avenue, Nanjing, Jiangsu 211166, China.

E-mail: [fchen@njmu.edu.cn](mailto:fchen@njmu.edu.cn)

Tel: 025-86869342

**Supplementary Figures**


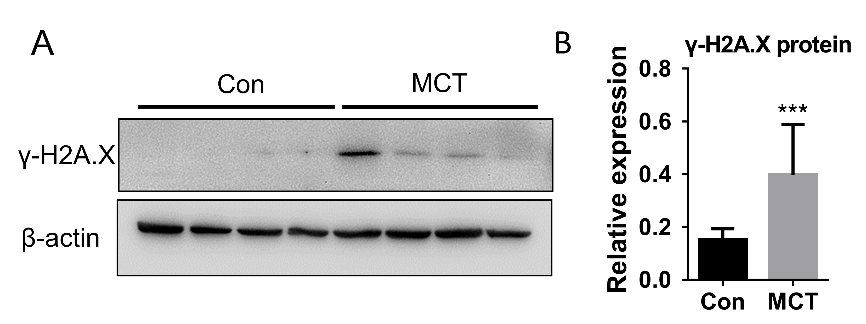


Figure S1. Expression of γ-H2A.X in MCT-induced PH rat model. A. Western blotting of γ-H2A.X from rat lung tissues of the contrl (n=4) and MCT group (n=4). B. Quantification of the protein expression of γ-H2A.X in the two groups. All data are shown as the mean ± SEM. Statistical significance compared to controls was assessed using the unpaired two-tailed Student’s *t*-test: *** *P*<0.001.


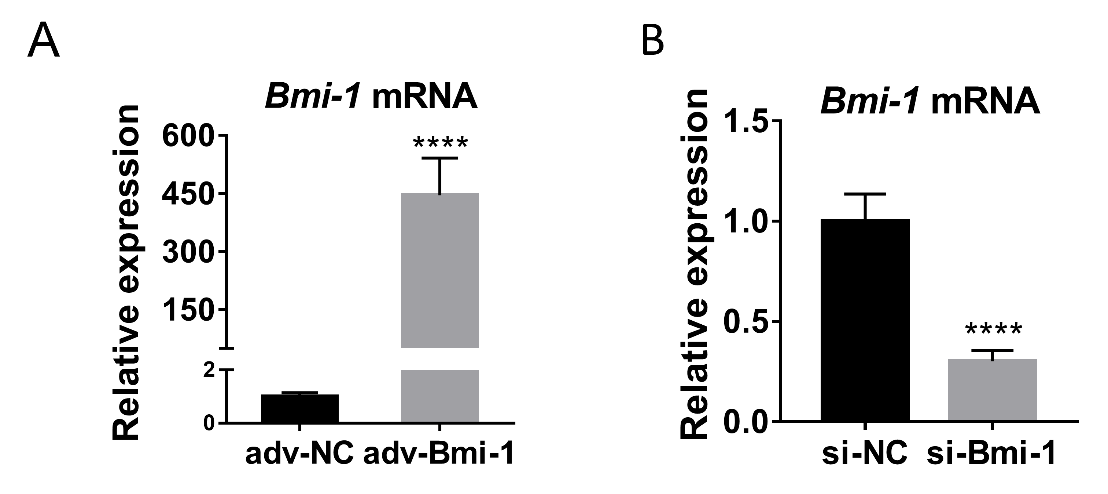


Figure S2. Transfection efficiency detection . The transfection efficiency of adv-Bmi-1 (A) and si-Bmi-1 (B) in HLFs. All data are shown as the mean ± SEM. Statistical significance compared to controls was assessed using the unpaired two-tailed Student’s *t*-test: *****P* < 0.0001.


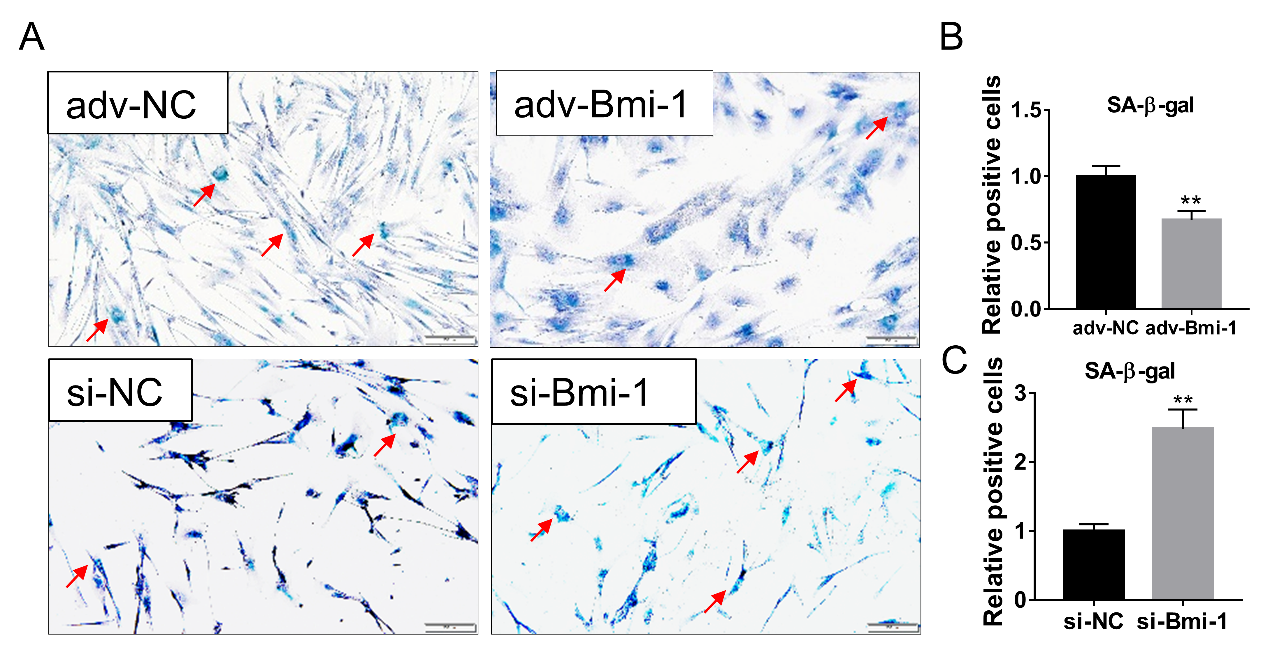


Figure S3. SA-β-gal staining of HLFs treated with adv-Bmi-1 and si-Bmi-1. A. Respresentitive images. Red arrows indicate the positively stained cells. B. The quantification of SA-β-gal positive cells are on the right. Three visual fields were randomly selected and the number of positive cells versus the total number of cells in each field was counted. All data are shown as the mean ± SEM. Statistical significance compared to controls was assessed using the unpaired two-tailed Student’s *t*-test: ***P*< 0.01.

**Raw data of Western Blotting**

1. Figure 2A





Left: Bmi-1, right: β-actin, Con VS. MCT: 3:4


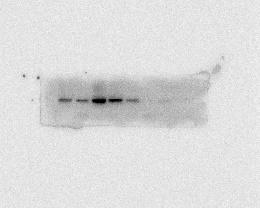


 Left: Bmi-1, right: β-actin, Con VS. MCT: 4:4


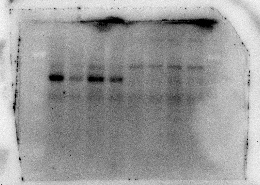


 Left: Bmi-1, right: β-actin, Con VS. MCT: 4:4

2. Figure 3A


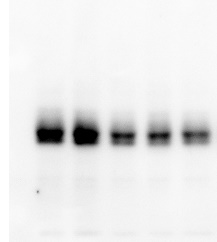

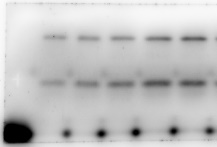

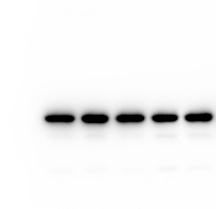
 Left: Bmi-1, middle: p16, right: GAPDH


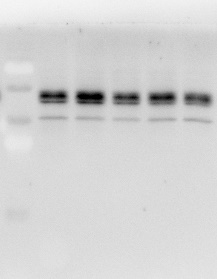

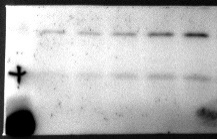


 Left: Bmi-1, middle: p16, right: GAPDH


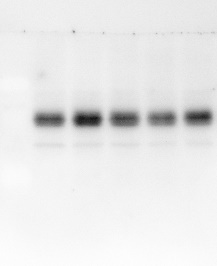

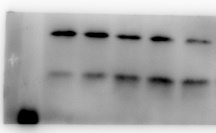


 Left: Bmi-1, middle: p16, right: GAPDH
